# Extracorporeal membrane oxygenation with continuous renal replacement therapy to treat metformin-associated lactic acidosis: A case report

**DOI:** 10.1097/MD.0000000000020990

**Published:** 2020-06-26

**Authors:** Ting Chen, Chunyan Zhu, Bao Liu

**Affiliations:** a Department of Critical Care Medicine, The Second People's Hospital of Hefei, Yaohai District; b Department of Critical Care Medicine, The First Affiliated Hospital of USTC, Division of Life Science and Medicine, University of Science and Technology of China, Hefei, Anhui, China.

**Keywords:** blood purification, continuous renal replacement therapy, extracorporeal membrane oxygenation, metformin-associated lactic acidosis

## Abstract

**Rationale:**

Metformin-associated lactic acidosis (MALA) is rarely encountered, but has a high mortality rate, Conventional treatments include hemodialysis or continuous renal replacement therapy (CRRT); however, when the disease progresses to end-stage, cardiac function is significantly inhibited, circulation cannot be maintained, CRRT cannot be tolerated, V-A extracorporeal membrane oxygenation (ECMO) may be the last treatment.

**Patient concerns:**

The study report a rare case of MALA in an elderly female patient at the age of 72 who was admitted to hospital because of nausea for 2 days, complicated with systemic fatigue.

**Diagnosis:**

MALA was cofirmed because of patient have increased lactic acid levels, blood pH <7.2, and a history of oral metformin intake.

**Interventions:**

Venoarterial ECMO (V-A ECMO) combined with CRRT was initiated when circulation was still not hold after intravenous fluids and 5% sodium bicarbonate were prescribed.

**Outcomes:**

V-A ECMO was then terminated after 48 hours when circulation was perserved, CRRT was discontinued when PH and lactic acid level were normal limited. etformin-associated lactic acidosis did not recur during 6 months follow-up.

**Lessons:**

The incidence of MALA is low, but mortality is very high. Intermittent hemodialysis or CRRT should be performed if the lactic acid level is persistently elevated. When severe circulatory dysfunction occurs and cardiac function is inhibited, V-A ECMO support should be performed immediately to maintain circulation, followed by CRRT, which may be the final measure to treat refractory MALA.

## 1 Introduction

Metformin is a commonly-used oral biguanide for the treatment of type 2 diabetes mellitus. Metformin is an inexpensive and effective drug that enhances insulin resistance and is widely prescribed in clinical practice.^[1]^ Nevertheless, metformin can also cause rare, severe, and fatal complications, such as lactic acidosis. The incidence of metformin-associated lactic acidosis (MALA) has been reported rarely, but the mortality rate is between 30% and 50%.^[2]^ Currently, no consensus has been reached regarding the diagnostic and treatment standards for MALA. The diagnosis is based mainly on the basic clinical manifestations of lactic acidosis and the presence of circulatory failure resistant to vasoactive drugs. Continuous renal replacement therapy (CRRT) is the primary treatment, but its use has been reported rarely. Additionally, extracorporeal membrane oxygenation (ECMO) combined with CRRT in the treatment of MALA has not been reported, to our knowledge. We report a patient with MALA and severe circulatory failure complicated by a wide QS arrhythmia, who was successfully treated using ECMO with CRRT.

## 2 Case report

**Figure 1:**
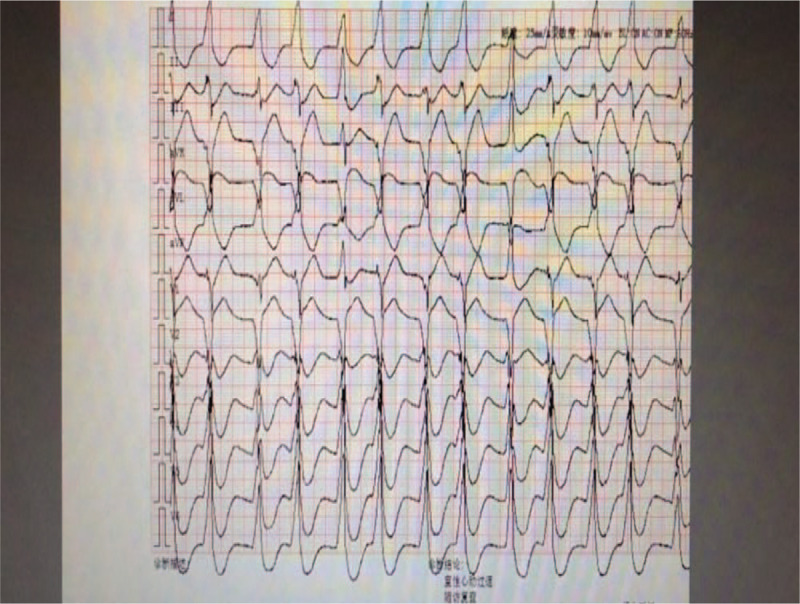
Electrocardiogram (ECG) showing a QRS of 132 ms.

**Figure 2:**
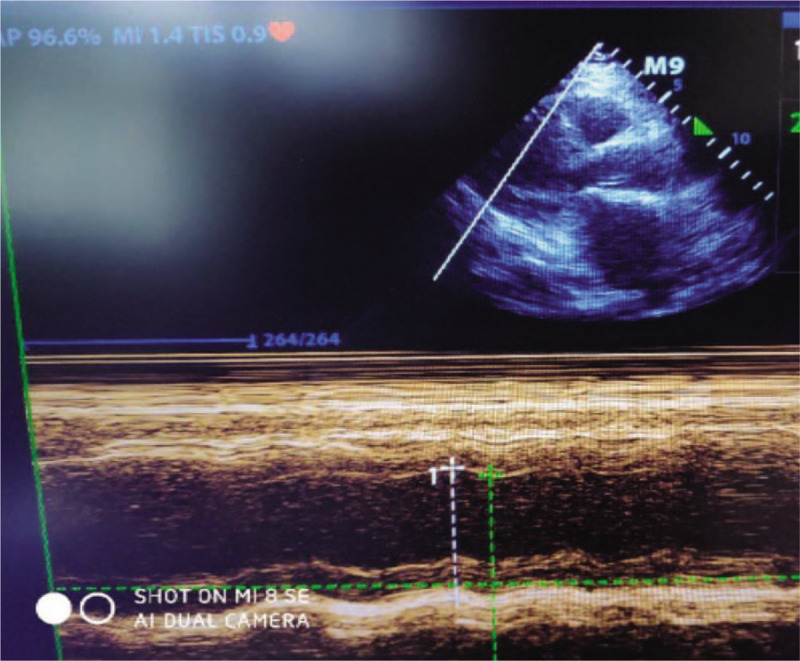
Echocardiogram demonstrated diffuse reduction of left ventricular wall motion, the left ventricular ejection fraction (EF%) was 23.5%.

**Figure 3:**
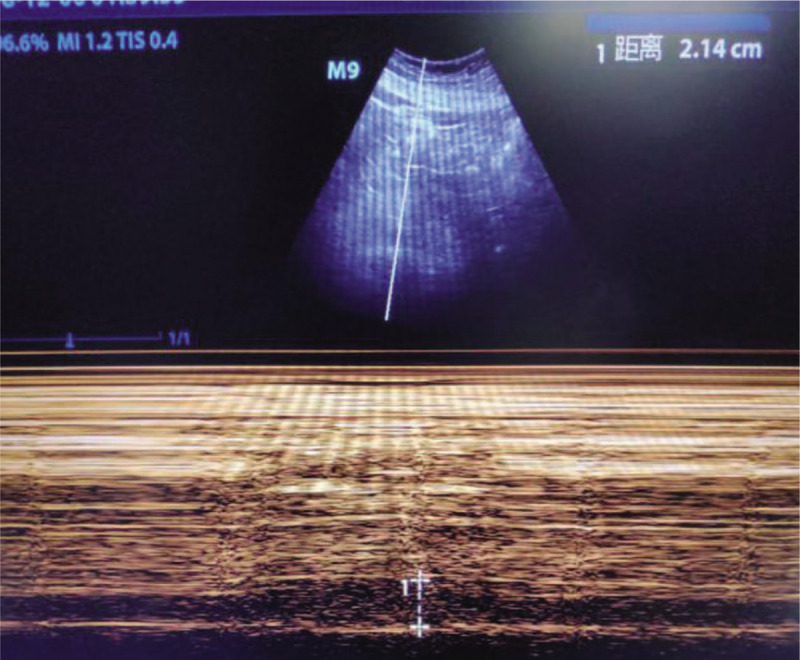
B-mode ultrasonography shows the end-inspiratory width of the inferior vena cava was 2.92 cm.

The patient was a 72-year-old woman who was admitted to our hospital with a history of 2 days of nausea complicated with systemic weakness. She had a history of type 2 diabetes mellitus and was taking oral metformin at a dose of 20 mg daily. In the emergency department, she was conscious, but had impaired mental status and cold limbs.Noninvasive arterial blood pressure (BP)was 62/34 mmHg, an electrocardiogram showed tachycardia with a wide QRS, blood gas analysis revealed a pH of 6.80, the concentration of arterial blood lactic acid was > 15 mmol/L, partial pressure of carbon dioxide was 14 mmHg, and the base excess was −26.5 mmol/L. The patient had deep respirations, and presented with nausea and vomiting and other gastrointestinal symptoms. Emergency treatment was initiated with 250 mL of 5% sodium bicarbonate,2.5 μg/kg/min of norepinephrine, and 1000 mL of normal saline. The patient was then transferred to the intensive care unit. One hour after admission, the patient had deteriorated. An electrocardiogram showed tachycardia with widening of the QRS wave (QRS: 132 ms; Fig. 1), blood gas analysis showed a pH of 6.93, blood lactic acid level was > 15 mmol/L, partial pressure of carbon dioxide was 13.2 mmHg, and her blood pressure was unchanged. Echocardiography demonstrated diffuse reduction of left ventricular wall motion, the left ventricular ejection fraction (LVEF) was 23.5% (Fig. 2), and the end-inspiratory width of the inferior vena cava was 2.14 cm (Fig. 3). Dobutamine at 1.0 μg/kg/min, a positive inotropic drug, was given, and 30 minutes later, the patient's LVEF had increased to 25.5%. However, her hemodynamics were even less stable, and we installed venoarterial extracorporeal membrane oxygenation (V-A ECMO) equipment (centrifugal pump, Maquet Rotaflow RF 32; Maquet Cardiopulmonary AG, Hirrlingen, Germany) at the bedside. The ECMO catheter (Duraflo; Edwards Lifesciences, Irvine, CA, USA) was inserted via the left femoral artery and right femoral vein. A 15-F catheter was selected for the femoral artery at a depth of 8 cm behind the lateral port, and a 19-F catheter was chosen for the femoral vein and was placed in the right atrium under B-ultrasound guidance. Finally, the catheters for the femoral artery and vein were connected with vascular access to the ECMO sheath to deliver adjuvant therapy to support cardiopulmonary function at a flow rate of 3.0 L/min. The speed of the ECMO pump was 2700 r/min, arterial pressure was 124 mm Hg, and venous pressure was −45 mm Hg. We used heparin for anticoagulation, and APTT was maintained at 45–55 s. CRRT was simultaneously connected in the continuous veno venous hemofiltration (CVVH) mode at a blood flow rate of 100 mL/min, which was gradually increased to 180 mL/min. The speed of the replacement fluid administration was 2000 ml/h, the ultrafiltration rate was 0 ml/h, the blood flow rate was 200 mL/h after 6 hour, and the speed of the replacement fluid delivery was increased to 3000 ml/h. Blood gas analysis at 2, 6, 12, and 24 hour demonstrated that the concentration of lactic acid declined to 8.3 mmol/L after 12 hour. An electrocardiogram revealed sinus rhythm, and cardiac color Doppler ultrasound at 48 hour showed an ejection fraction of 52.4%. CRRT treatment was then terminated, and the ECMO rotation speed was decreased to 1000 *r*/min, at which time, the patient's mean arterial pressure was 75 mmHg, and central venous oxygen saturation was 78%. The ECMO flow rate was gradually decreased to 1.0 L/min, and the ECMO equipment was then withdrawn, and the femoral artery and vein catheters were removed. Tracheal intubation was discontinued on the 5th day, and the patient was transferred to the internal medicine emergency department on the 6th day and discharged on the 10th day. She was followed up by telephone at 1, 3, and 6 months. Following treatment, the patient was stabilized and returned to her normal life. MALA did not recur during the postoperative follow-up.

## 3 Discussion

MALA has a low incidence, but a high mortality rate. Although blood purification can improve the clinical prognosis in patients with MALA, the mortality rate remains approximately 20%. For patients with a blood pH < 6.9 and severe MALA, the mortality rate is as high as 83%.^[3]^ Patients with MALA lack specific clinical manifestations^[4]^; however, gastrointestinal symptoms are the primary signs, namely, nausea, vomiting, abdominal distension, and diarrhea. In the advanced stage, systemic weakness, hypotension, changes in mental status, and even serious arrhythmias may occur. Our patient presented with all of these symptoms. The possibility of MALA should be considered if patients have increased lactic acid levels, blood pH < 7.2, and a history of oral metformin intake. Blood gas analysis should be performed immediately in patients with these findings.

ECMO describes extracorporeal circulation equipment with a lung membrane and centrifugal pump as the core components, and can save time when treating reversible heart and lung diseases.^[5]^ With progressive developments in ECMO, this approach has been used in a wider range of conditions, from initial cardiopulmonary function support after major cardiac surgery and in patients with end-stage structural lung disease waiting for lung transplantation to various critical diseases, such as cardiopulmonary resuscitation, acute myocardial infarction, severe fulminant myocarditis, and poisoning. ECMO plays an increasingly pivotal role in clinical practice. In this report, the patient presented with significantly suppressed cardiac function (the lowest LVEF 23.5%), circulatory dynamic disorders (BP 62/34mm Hg with norepinephrine 2.5 μg/kg/min and dobutamine 1.0 μg/kg/min, QRS of wave 132ms), and V-A ECMO indications caused by MALA, which was a reversible disorder. Moreover, the patient's circulatory dynamics improved significantly after ECMO treatment. Thirty minutes after ECMO, we were able to decrease the dose of vasoactive drugs (norepinephrine at a dose of 1.0 μg/kg/min), and the patient's blood pressure had increased to 90/52 mm Hg.

ECMO treatment has been used in an increasing number of patients worldwide, and the ECMO indications have increased correspondingly. ECMO has been proven effective when treating fulminant myocarditis.^[6]^ Maejima et al^[6]^ demonstrated that ECMO has unique advantages when treating severe fulminant myocarditis, and the long-term survival rate is > 75%. Riera et al^[7]^ proposed that although the clinical efficacy of ECMO in treating septic shock remains uncertain, ECMO is effective for specific cases. Our patient was successfully treated using ECMO. The first benefit of ECMO is that it can rapidly improve respiratory, cardiac, circulatory, and other functions; thus, providing a treatment opportunity for subsequent CRRT treatment. The second benefit is that ECMO provides an oxygen supply to reduce the risk of anaerobic hydrolysis. The optimal time for ECMO and adjuvant therapy remains controversial. In most circumstances, V-A ECMO treatment should be initiated immediately for patients with MALA-induced circulatory dysfunction and heart involvement, low perfusion, and persistent aggravation of tissue hypoxia, even after receiving high-dose vasoactive drugs. The specific indications for V-A ECMO are^[5]^:

(1) low cardiac output syndrome (cardiac index (< 2 L/m2/min) lasting > 3 h and/or accompanied by metabolic acidosis (base excess > −5 mmol/L));
(2) cardiogenic shock nonresponsive to drugs and aortic balloon counter-pulsation;
(3) cardiac arrest; and
(4) severe arrhythmia induced by metformin.

The optimal rescue opportunity may be missed when the base excess is > −5 mmol/L for > 12 hour, lactic acid level is > 10 mmol/L for > 10 hour, and urine volume is > 0.5 ml/kg/h for > 12 hour. In this report, the timing of V-A ECMO was appropriate because the patient presented with a base excess > −5 mmol/L and a lactic acid level > 10 mmol/L, which are consistent with the indications for V-A ECMO.

Intermittent hemodialysis or CRRT is considered an emergent measure for MALA. Lactic acid and metformin are small molecular substances with molecular weights of 90.08 g/mmol and 165 g/mmol, respectively, and are water-soluble substances that are mainly eliminated through a dispersion mechanism. Therefore, both intermittent hemodialysis and CRRT can remove lactic acid and metformin. Intermittent hemodialysis is suitable for patients with stable hemodynamics. CRRT exerts less impact on hemodynamics compared with intermittent hemodialysis, which is applicable for patients with circulatory dynamic disorders.^[8,9]^ In this report, the patient had severe shock. The dose of norepinephrine was > 2.0 ug/kg/min, the QRS wave was widened, the lowest pH was 6.82, and there was significant decrease in the LVEF on admission. The patient was suspected to have severe acidosis, which inhibited myocardial metabolism, affected the myocardial diastolic and systolic functions, and was a CRRT contraindication. Therefore, she received V-A ECMO with CRRT, (V-A ECMO preserved circulatory dynamics,CRRT scavenged lactate and metformin)and we connected CRRT in ECMO with anterior membranous connection after ECMO pump using the CVVH mode. A small dose of ultrafiltration solution was initially administered to minimize the influence of CVVH on circulatory function. Forty-eight hours after ECMO with CRRT, the patient's circulation stabilized, the lactic acid level decreased to normal, and her cardiac ejection fraction had increased significantly (LVEF from 23.5% to 52.4%), likely because ECMO maintained partial circulatory function, provided an oxygen supply, mitigated tissue hypoxia, and provided opportunities for CRRT treatment CRRT eliminated the metformin and lactic acid, which destroyed the vicious negative cycle occurring in the circulatory system.

In conclusion, the incidence of MALA is low. Retrospective studies have been performed and case reports describing MALA have been published, but randomized controlled studies are still lacking. No consensus has been achieved regarding the diagnostic criteria for MALA in clinical practice.^[10,11]^ Consequently, it is extremely important to improve the diagnosis of MALA by clinicians, especially emergency medicine physicians. Bicarbonate infusion is a recommended option for MALA; however, intermittent hemodialysis or CRRT should be considered if the lactic acid level is persistently increased. Finally, V-A ECMO support should be performed immediately when severe circulatory dysfunction occurs and cardiac function is significantly suppressed, to maintain circulatory function, followed by CRRT, which is the final intervention for MALA treatment.

## Acknowledgment

We thank all of the medical staff of Hefei Second People's Hospital for their support and Bao Liu for English language editing.

## Author contributions

Ting Chen and Chunyan Zhu designed the paper and wrote the first draft. All authors participated in drafting and reviewing the manuscript. All authors read and approved the final manuscript.

**Data curation:** Bao Liu.

**Formal analysis:** Bao Liu.

**Resources:** Bao Liu.

**Supervision:** Bao Liu.
